# Confocal Raman Spectroscopy for Assessing Bioequivalence of Topical Formulations

**DOI:** 10.3390/pharmaceutics15041075

**Published:** 2023-03-27

**Authors:** Fotis Iliopoulos, Chun Fung Tang, Ziyue Li, Annisa Rahma, Majella E. Lane

**Affiliations:** 1 Department of Pharmaceutics, UCL School of Pharmacy, 29–39 Brunswick Square, London WC1N 1AX, UK; 2 Pharmaceutics Department, School of Pharmacy, Institut Teknologi Bandung, Bandung 40132, Indonesia

**Keywords:** Confocal Raman Spectroscopy, bioequivalence, topical formulations, skin delivery

## Abstract

The evaluation of bioequivalence (BE) for topical dermatological drug products is challenging, and there has been significant interest from regulatory authorities in developing new BE methodologies in recent years. Currently, BE is demonstrated by comparative clinical endpoint studies; these are costly and time-consuming and often lack sensitivity and reproducibility. Previously, we reported excellent correlations between in vivo Confocal Raman Spectroscopy in human subjects and in vitro skin permeation testing (IVPT) with the human epidermis for skin delivery of ibuprofen and a number of excipients. The aim of the present proof-of-concept study was to evaluate CRS as a method to assess BE of topical products. Two commercially available formulations, Nurofen Max Strength 10% Gel and Ibuleve Speed Relief Max Strength 10% Gel, were selected for evaluation. Delivery of ibuprofen (IBU) to the skin was determined in vitro and in vivo by IVPT and CRS, respectively. The formulations examined were found to deliver comparable amounts of IBU across the skin over 24 h in vitro (*p* > 0.05). Additionally, the formulations resulted in similar skin uptake values measured with CRS in vivo, either at 1 h or 2 h after application (*p* > 0.05). This is the first study to report the capability of CRS for the demonstration of BE of dermal products. Future studies will focus on the standardisation of the CRS methodology for a robust and reproducible pharmacokinetic (PK)-based evaluation of topical BE.

## 1. Introduction

The development of efficient methods for the evaluation of topical bioequivalence (BE) has been reported to promote patient access to high-quality generic products and is generally associated with economic and societal benefits [1]. Formulations, administered at the same doses under similar conditions in an appropriately designed study, are considered bioequivalent when the rate and extent to which the active ingredient becomes available at the site of action are not significantly different [2]. The demonstration of BE typically involves the assessment of the efficacy of generic formulations compared with a reference drug product. For such determinations, it is critical that the methods used are sensitive and reproducible [3].

To date, only limited approved methods are available for evaluating dermal BE. For drugs that are intended to act systematically, in vivo pharmacokinetic (PK) studies via blood sampling may be conducted. For topically acting compounds, determining drug concentrations inside the skin has been challenging, and plasma concentrations may not represent the amounts of drug at or near the site of action, i.e., the stratum corneum (SC), the viable epidermis, or the deeper cutaneous tissues. For such compounds, BE is typically evaluated by comparative clinical endpoint studies [4]; however, this approach is expensive and time-consuming and is also reported to have lower efficiency compared with PK studies [5]. An alternative BE method is based on using pharmacodynamic (vasoconstriction) endpoint measures; this approach is only feasible for topical glucocorticoid drug products. 

In addition to the in vivo BE methodologies, in vitro techniques that correlate with in vivo data may also be utilised. Specifically, the in vitro permeation test (IVPT) in excised human skin has been accepted as an appropriate method for determining percutaneous absorption. The utility of IVPT in BE testing of topical formulations has been reported in numerous studies [6,7,8,9]. Recently, Shin et al. [10] conducted IVPT studies in dermatomed human skin to investigate BE of four commercially available creams containing acyclovir 5%. A dose of 15 mg/cm^2^ was applied to the skin, and the efficacy of the various products was investigated. Comparisons were made in terms of maximum flux (J_max_) values and the total amounts of drug permeated, expressed as the area under the curve (AUC) of the permeation profiles. The researchers reported that the IVPT method confirmed the BE of positive controls (reference vs. itself; test vs. itself). Additionally, it was possible to delineate the different efficacies of the formulations with respect to drug delivery. However, despite the efficacy of the IVPT model for BE determination, the experimental conditions of such studies cannot accurately mimic real-life conditions and BE documentation may normally not be supported solely by IVPT results, with certain exceptions [11]. Thus, to date, there remains a need for new approaches that have the capability to determine BE of generic topical products [12,13]. The development of alternative BE methodologies has also been a major goal for the regulatory authorities in the last decades [1]. 

Confocal Raman Spectroscopy (CRS) has been widely used in skin research [14,15]. Harnessing inelastic light scattering, CRS is non-invasive and enables the real-time monitoring of penetration of topically applied substances across the skin. The detection of substances is based on the Raman spectra recorded by the instrument for each focal plane and subsequent data processing for the identification of distinct spectral signatures of the selected molecules under investigation. The potential of CRS for BE determinations was previously proposed [16,17]; however, the utility of the method for BE had been limited due to a lack of quantification capabilities. Recently, Caspers et al. [18] reported a novel application of CRS for quantitative analysis of the amounts of compounds that penetrate the skin. The quantitation of total skin uptake was performed by the calculation of the area under the depth profile curves (AUC) for the SC thickness. The findings obtained by this approach were subsequently validated against the IVPT model [19,20]. Overall, excellent correlations were found following the linear regression of the cumulative permeation of the drug in vitro and the corresponding skin uptake values measured by CRS in vivo. The active ingredients evaluated were niacinamide and ibuprofen (correlation coefficient R^2^ = 0.94 and 0.90, respectively). Additionally, the cumulative amounts of various excipients, namely propylene glycol, dipropylene glycol, and tripropylene glycol, which permeated in vitro, were also found to correlate well with the amounts that were taken up by the SC in vivo (R^2^ = 0.82). These studies confirm that CRS is a powerful technique for profiling drug and vehicle delivery to the skin; however, to date, the capability of CRS to evaluate BE of products has not been examined.

In the present work our aims were (i) to examine ibuprofen delivery from two commercial products using both IVPT studies and CRS in vivo studies and (ii) to assess the CRS method as a novel approach to demonstrate BE for topical drug products. Ibuprofen (IBU) has been available for over-the-counter purchase for many years in a range of different formulations [21]. The formulations examined, Nurofen Max Strength 10% Gel and Ibuleve Speed Relief Max Strength 10% Gel (marketing authorisation numbers PL 10972/0089 and PL 00173/0176, respectively), have been approved by the UK national regulatory agency and the Medicines and Healthcare Products Regulatory Agency (MHRA) [22,23,24,25].

## 2. Materials and Methods

### 2.1. Materials

IBU, methanol, Brij^®^ O20, or Polyoxyethylene (20) oleyl ether were provided by Sigma-Aldrich (Dorset, UK). All other materials were of analytical grade unless otherwise specified. The formulations tested, Nurofen Joint & Back Pain Relief Max Strength 10% Gel (B/N: P607; Mercury Pharma Group Ltd., London, UK) and Ibuleve Speed Relief Max Strength 10% Gel (B/N: X1032; Diomed Developments Ltd., Herts, UK), were purchased from a local pharmacy. Excised abdominal human skin from one single donor was obtained from cosmetic surgery procedures with informed consent and institutional ethical approval (Research Ethics Committee reference 07/H1306/98). Human tissues were preprepared as described in a previous paper [19].

### 2.2. Methods

#### 2.2.1. HPLC Analysis

An HP1100 device (Hewlett-Packard, Palo Alto, CA, USA) was used for IBU analysis, and data were acquired and processed with ChemStation^®^ for LC 3D, Rev. B. 04.03 software (Agilent Technologies, Santa Clara, CA, USA). The determination of IBU was performed using the analytical method reported previously [20], with slight modifications. Specifically, analysis was conducted using a Luna^®^ 5 µm C8(2) 100 Å, LC column (Phenomenex, Macclesfield, UK) with dimensions 150 × 4.6 mm. Methanol:water (80:20) and TFA 0.1% (*v*/*v*) was prepared as the mobile phase. The detection wavelength was 222 nm, the column temperature was 30 °C, and the sample injection volume was 10 μL. The flow rate was set to 1 mL/min. International Conference on Harmonisation (ICH) guidelines Q2 (R1) were followed for method validation and IBU eluted at 4 min [26]. Values of 0.22 µg/mL and 0.66 µg/mL were obtained for the limit of detection (LOD) and the limit of quantification (LOQ), respectively.

#### 2.2.2. IVPT and Mass Balance Studies

The IVPT studies were conducted according to OECD guidelines and are described in detail in our previous publication [27,28,29]. Impedance measurements were used to confirm the integrity of all skin samples. All experiments were conducted with a skin temperature of 32 °C under unoccluded conditions. Permeation experiments were conducted for 24 h, with a regular sampling of the receptor compartment [19,20,29]. The number of replicate experiments was *n* = 5. The formulations tested were Nurofen Max Strength 10% Gel (Nurofen) and Ibuleve Speed Relief Max Strength 10% Gel (Ibuleve). The pharmaceutical compositions of the two formulations tested are provided in Table 1. 

After the permeation study, the skin surface was washed using 80% (*v*/*v*) methanol in water with five consecutive 1 mL volumes and dried with a cotton swab. The skin membrane was then placed in an Eppendorf^®^ tube with 1 mL of methanol for extraction. The samples were mixed using a Vortex mixer (IKA^®^ vortex mixer genius 3, VWR International Limited, Leicestershire, UK) and were centrifuged for 1 min at 13,000 rpm at 32 °C to ensure that all skin pieces were fully immersed in the solution. Subsequently, they were placed in an orbital mini shaker for 5 h (VWR International Limited, Leicestershire, UK), processed as reported in our previous publications [19,20,29] and analysed by the validated HPLC method.

#### 2.2.3. Confocal Raman Spectroscopy In Vivo

The details of the CRS device and data collection have been described in detail elsewhere [19,20]. Research ethics committee approval was obtained for this study (RN 0234/0624). Five volunteers (3 male, 2 female, 2 Caucasian, 3 Asian, ≥21 years of age) were recruited, and the site of analysis was the forearm. A finite dose, 10 μL/cm^2^, of 10% (*w*/*w*) IBU formulations was applied over an area of 3.8 cm^2^ using a positive displacement pipette (Eppendorf^®^ Multipipette Plus, Hamburg, Germany) and were spread evenly over the marked area with a disposable inoculating loop (VWR International Limited, Leicestershire, UK). The formulations tested were Nurofen and Ibuleve, as for the IVPT studies. After either 1 h or 2 h, any excess of formulation was removed from the skin surface using (a) one cotton bud soaked in water with 6 % (*w*/*v*) polyoxyethylene (20) oleyl ether (Brij^®^ O20) followed by (b) one cotton bud soaked in distilled water and finally (c) the skin was swabbed with one dry cotton bud. The cleaning procedure was performed to ensure that no amounts of IBU were left on the skin surface. Brij^®^ O20 is a non-ionic Oleth-surfactant. It is widely used as a cleansing ingredient in cosmetic products, and its safety has been confirmed by the Cosmetic Ingredient Review (CIR) Expert Panel [30]. 

Prior to application of formulations, the marked areas on the forearm of each volunteer were measured with the 785 nm excitation laser (FP region, 10 s exposure, 2 μm steps 28 μm depth), and these readings served as the baseline. For the determination of SC thickness [14], untreated areas of the skin were measured with the 690 nm laser (HWN region, 2 s exposure, 4 μm steps, and 40 μm depth). 

#### 2.2.4. Data Analysis

RiverICon V 3.0.130327 software (RiverD International B.V., Rotterdam, The Netherlands) was used for data collection. Skin Tools 2.0 (RiverD International B.V., Rotterdam, The Netherlands) software was used for data processing. Calibration was considered successful when the signal-to-noise ratio was above 30. The determination of the API amounts across the skin was made by a least squares fitting algorithm, as previously developed and described by Caspers et al., using the spectral information of the FP region [31]. Drug signal profiles were corrected with untreated baseline skin profiles. To analyse two groups and ≥3 groups, the independent-samples *t*-test and one-way analysis of variance (ANOVA) with Tukey’s post hoc test were used, respectively. A *p* < 0.05 was considered statistically significant. R (ver: 4.1.0) and RStudio (ver: 1.4.1717) were used for all statistical analyses.

## 3. Results and Discussion

### 3.1. IVPT and Mass Balance Studies

**Figure 1:**
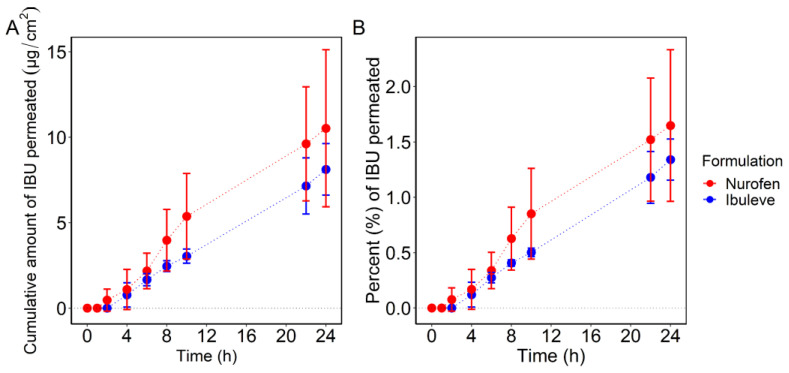
Permeation profiles of IBU over time for the commercially available formulations, Ibuleve and Nurofen, in human epidermis, expressed as (**A**) cumulative amounts (μg/cm^2^) and (**B**) percentages (%) of the dose applied (*n* = 5; mean ± SD).

The permeation profiles of IBU over 24 h, expressed as cumulative amounts (μg/cm^2^) and percentages (%) of the dose applied are shown in Figure 1.

**Figure 2:**
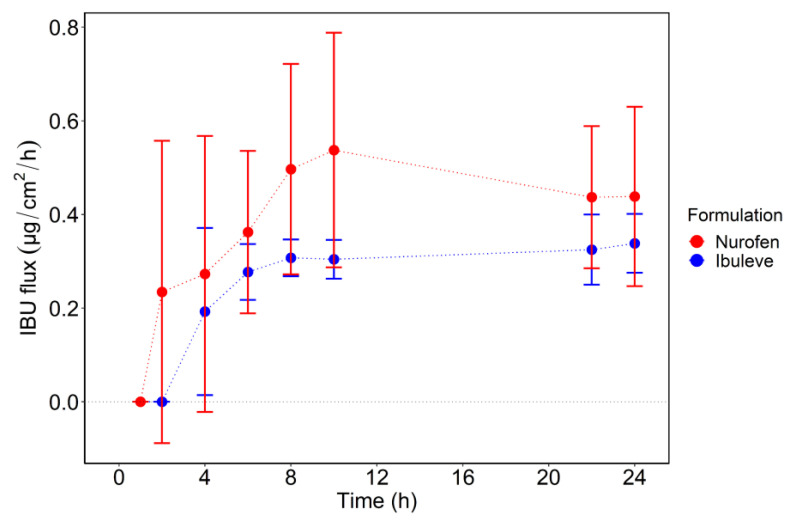
Estimated flux profiles of IBU (μg/cm^2^/h) through human epidermis following application of the commercially available formulations, Ibuleve and Nurofen (*n* = 5; mean ± SD).

The two formulations examined, Ibuleve and Nurofen, delivered comparable amounts of IBU across the skin over 24 h, i.e., 8.1 ± 1.5 µg/cm^2^ and 10.5 ± 4.6 µg/cm^2^, respectively (*p* > 0.05). The cumulative amounts of IBU that permeated through human skin at the earlier time points (4 h, 6 h, 8 h, 10 h, and 22 h) were also comparable for both formulations (*p* > 0.05). With regards to the percentages of IBU that permeated over 24 h, similar values were found for Ibuleve and Nurofen, namely 1.3 ± 0.2% and 1.6 ± 0.7% of the doses applied (*p* > 0.05), respectively. For Nurofen, IBU was detected in the receptor compartment at 2 h (0.5 ± 0.7 μg/cm^2^; 0.1 ± 0.1% of dose applied), while for Ibuleve, permeation of IBU was not apparent until 4 h (0.8 ± 0.7 μg/cm^2^; 0.1 ± 0.1% of the dose applied). With regards to the rate of drug permeation, the maximum flux (J_max_) values of IBU were similar for Ibuleve (0.3 ± 0.1 μg/cm^2^/h) and Nurofen (0.5 ± 0.2 μg/cm^2^/h, *p* > 0.05). For Ibuleve, IBU flux showed an increase in the first 8 h to reach a maximum value of 0.3 ± 0.1 μg/cm^2^/h and remained relatively constant until the end of the experiment. For Nurofen, IBU flux reached a maximum value of 0.5 ± 0.2 μg/cm^2^/h at 10 h, which was then followed by a decrease up to 24 h (0.4 ± 0.2 μg/cm^2^/h). The IBU flux profiles (μg/cm^2^/h) from the two gels are shown in Figure 2.

To our knowledge, there are no previous studies that have reported IBU permeation in human skin from Ibuleve and Nurofen 10% (*w*/*w*) gel formulations. Recently, Pradal [32] examined the skin absorption of IBU from six commercially available topical products, including two 10 % (*w*/*w*) gel formulations. These products were labelled as Ibu-5 and Ibu-6 and were reported to have the same qualitative composition as Nurofen and Ibuleve, respectively; however, the marketed names were not disclosed by the author. IBU absorption across the skin was examined using finite dose IVPT studies in human skin (10 mg/cm^2^) over a 24 h period. The geometric means for the cumulative amounts of IBU permeated were 15.4 μg/cm^2^ for Ibu-5 and 25.3 μg/cm^2^ for Ibu-6, respectively. These values are greater than the cumulative permeation of IBU from the two formulations examined in the present study. Here, geometric means were calculated as 8.0 and 9.8 μg/cm^2^ for Ibuleve and Nurofen, respectively. These differences in IBU permeation may be due to (i) different quantitative compositions or preparation methods for the formulations tested; (ii) the different sample sizes of the studies (six replicates from one donor vs. two replicates from six donors). 

**Figure 3:**
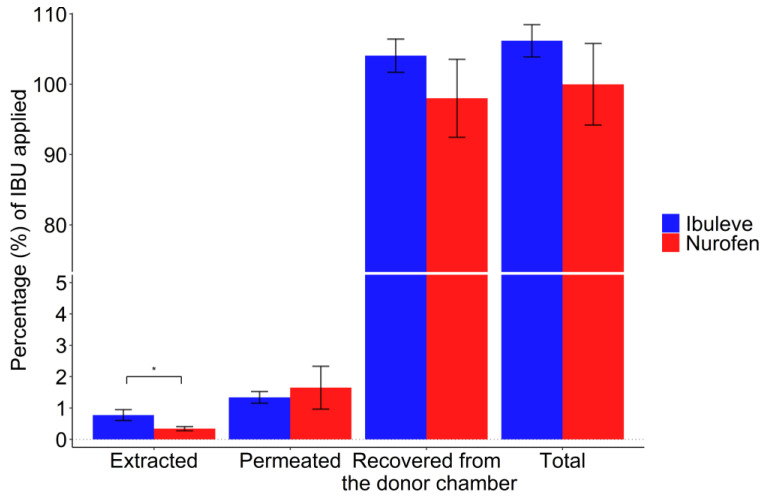
Percentage (%) recovery values of IBU for the commercially available formulations, Ibuleve and Nurofen, following mass balance studies in the human epidermis (*n* = 5; mean ± SD, * *p* < 0.05).

The results of the mass balance studies for IBU formulations are shown in Figure 3.

The percentages of IBU recovered from the skin surface for Nurofen and Ibuleve were 98.0 ± 5.6% and 104.1 ± 2.4% of the dose applied, respectively (*p* > 0.05). The high percentages of IBU deposited on the skin surface might be attributed to drug crystallisation following vehicle depletion via skin uptake and/or evaporation. Both gels contain a volatile solvent, either isopropyl alcohol (Nurofen) or industrial methylated spirit, typically ethanol 95% (*v*/*v*) [33] in the case of Ibuleve. Previous studies have reported that these solvents evaporated completely within 6 min following the application of finite dose volumes [20,29]. As shown in Table 1, depletion of alcohol from the skin may leave a residual phase consisting mainly of a polymer, either carbomers or hydroxyethyl cellulose, and water. Under these conditions, drug crystallization on and inside the skin may occur, especially considering the low solubility of IBU in purified water, 0.1 mg/mL [34]. Drug crystallisation is known to negatively affect drug topical and transdermal bioavailability, as previously described in detail in several studies [35,36,37]. With regards to the amounts of IBU extracted from inside the skin, Ibuleve deposited 0.8 ± 0.2% of the applied dose in the epidermis. This value was significantly higher than the corresponding value for Nurofen (0.3 ± 0.1%, *p* < 0.05). Overall, the total recovery of IBU after mass balance studies was within the acceptable range of 90–110% [27,28].

### 3.2. Confocal Raman Spectroscopy In Vivo

**Figure 4:**
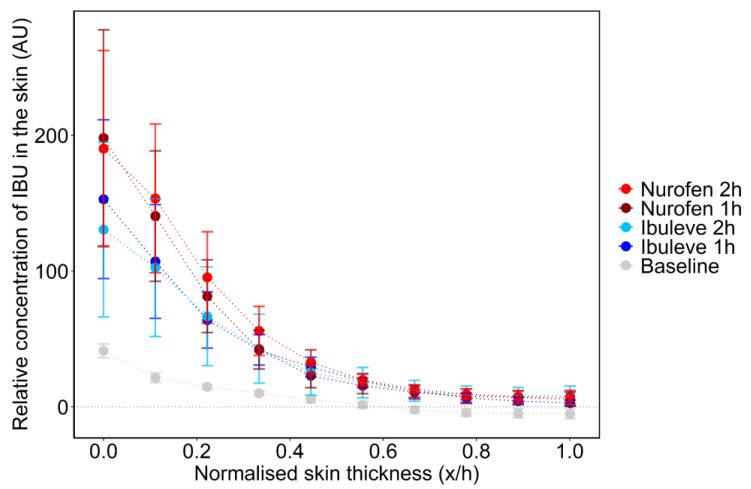
Depth profiles of IBU (AU) across the human volar forearm skin as a function of distance to the skin surface following a 1 h and 2 h application of formulations in vivo (mean ± SEM of 4 subjects; *n* = 8 replicates per subject).

The average SC thickness of the volunteers was 18.0 ± 0.8 μm, estimated as reported in previous studies [14]. This value was used to normalise the distance to the skin surface [16,38]. The IBU depth profiles across the volar forearm skin of the volunteers for the two formulations are shown below in Figure 4.

The signal intensity values of IBU (AU) at each depth examined were similar for both formulations either 1 h or 2 h post-application (*p* > 0.05). At 1 h, no IBU was detected at depths greater than 14 µm. This depth corresponded to an average depth interval of 0.8 ± 0.1 x/h of the estimated SC thickness. For Ibuleve, IBU signal intensity values across the SC after 1 h ranged from 126.6 ± 52.4 AU at the skin surface to 6.0 ± 2.7 AU at a depth of 0.8 x/h. The respective values detected for Nurofen ranged from 221.7 ± 66.0 to 12.1 ± 5.9 AU. At 2 h; the signal intensity of IBU at 1.0 x/h SC depth interval was 8.8 ± 5.3 AU and 9.4 ± 4.7 AU for Ibuleve and Nurofen, respectively. For either Ibuleve or Nurofen, the values of IBU Raman signal intensity measured at each depth at 1 h did not differ significantly compared with the corresponding values obtained at 2 h (*p* > 0.05). 

**Figure 5:**
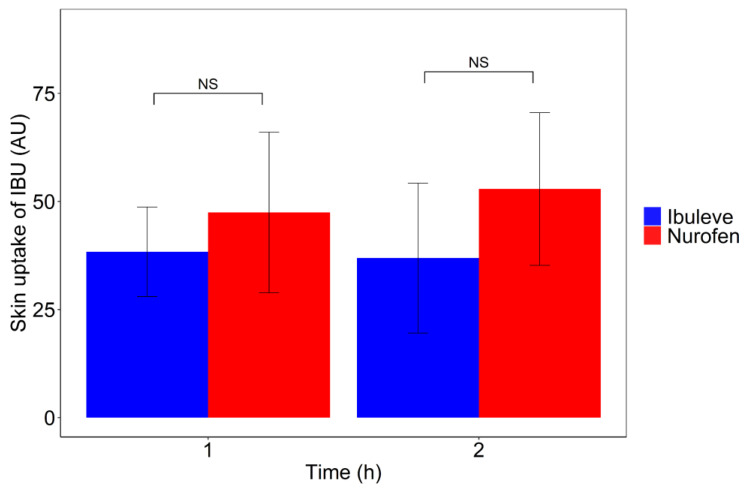
Area under the depth profile curve (AUC) values of IBU in the SC following a 1 h and 2 h application of formulations in vivo (mean ± SEM of 4 subjects; *n* = 8 replicates per subject).

With regards to the total amount of IBU that penetrated the SC, the area under the depth profiles curves (AUC) was used as a measure of drug uptake for each formulation, as reported previously [18,38,39,40]. The AUC values measured after a 1 h application were 36.9 ± 17.4 and 47.4 ± 18.6 AU for Ibuleve and Nurofen, respectively. These values were not statistically different from each other (*p* > 0.05). With regards to the 2 h application time interval, the estimated AUC values were also similar for both Ibuleve and Nurofen, with values of 38.4 ± 10.4 and 52.9 ± 17.7 AU, respectively (*p* > 0.05). The total amount of drug in the SC measured by CRS has been previously found to correlate with the cumulative amount of drug permeated measured with IVPT studies [19,20]. In the present study, the two products resulted in comparable amounts of IBU permeation in vitro (IVPT; Figure 1) as well as comparable skin uptake, estimated by the AUC values of IBU signal intensity determined with CRS in vivo (Figure 5).

Relevant guidance of the FDA and the EMA [41,42] recommends that BE between products shall be determined based on statistical comparisons of selected PK metrics that reflect the rate and extent of drug permeation. Typically, BE is established when the anti-log of the calculated confidence interval for the ratio of the population geometric means lies within the acceptable range of 80–125%. For IVPT studies, the cumulative amount penetrated across the duration of the study (Q24) has been reported to be analogous to the clinical area under the concentration–time curve, historically used in the case of plasma PK determinations [10,43,44]. Here, the difference between the natural log transformed geometric mean values of Q24 found for Nurofen and Ibuleve was 0.19. This value lies within the regulatory limit of ln(0.8) to ln(1.25), i.e., from −0.22 to 0.22, indicating that the formulations were equivalent for this donor. These calculations were based on data from one donor, and therefore the calculations of confidence interval values normally estimated when taking the average of geometric means for all subjects is not applicable for this IVPT study. Regarding CRS, the mean of the log-transformed average of the ratio of the geometric mean AUC values at 1 h across 4 subjects was found to be −0.13. This value is within the pre-set regulatory BE limits; however, it should be noted that the high inter-subject variability observed (90% confidence interval (CI): −1.5, 1.8) suggests that a greater number of experiments is required for a definitive BE determination. This variability is further denoted at the 2 h time point, where the mean difference of the log-transformed average of the geometric means was −0.4 (90% CI: −3.5, 2.6). The observed variability in the data may be attributed to inter-subject variability, as previously reported in the literature [45,46,47]. The high variability associated with skin permeation of compounds has been reported in the literature, and novel applications for statistical BE approaches, e.g., the scaled average BE approach, has been explored for potentially widening the BE limits [10]. For CRS to be taken forward as an appropriate methodology for BE determination, it is critical to understand the degree of data variability. Clearly, a full-scale PK study with multiple donors and additional time points is needed for a PK-based validation of the proposed methodology. Additionally, further studies using a wide range of drug substances and drug products are required to support the capability of CRS to assess the efficacy of products other than those tested in the present study. However, the present findings together with recent in vitro—in vivo correlation studies [17,19,20,38] demonstrate the capability of CRS to evaluate the BE of commercial formulations in vivo. The standardization of the procedure as well as the inclusion of appropriate power simulation analysis for an a priori determination of the time intervals and number of donors required is currently under development. It is important to note that the use of CRS for assessing BE of topical formulations is not applicable for all compounds. The main prerequisite for measurement by CRS is that the molecule of interest should possess spectral features with sufficient intensity, in which the signals can be differentiated from that of the skin components.

## 4. Conclusions

This is the first study to examine the utility of CRS for the assessment of BE between topical formulations in vivo. Permeation of IBU from two marketed IBU gel formulations was investigated using finite dose IVPT and CRS studies. The commercial formulations were found to promote similar IBU delivery across the skin in vitro over 24 h (*p* > 0.05). Additionally, the mean AUC values of IBU estimated by the CRS depth profiles across the SC were comparable for the two formulations at every time point examined, 1 h and 2 h (*p* > 0.05). These time points represented a common duration of exposure for pain-relief gel formulations in real life. In future work, experiments that can mimic conditions of repeated application of formulations will also be considered. Our findings support the application of the CRS technique and underline the potential of the method to demonstrate topical BE. Studies with additional formulations and drug substances spanning a range of different dosage forms and physicochemical properties, as well as a greater number of time points and subjects, are ongoing to evaluate this approach as a novel PK-based methodology for the determination of topical BE.

## Figures and Tables

**Table 1:** Ingredients of the topical formulations, Ibuleve and Nurofen.

Ibuleve Speed Relief Max Strength 10% Gel	Nurofen Max Strength 10% Gel
IBU	IBU
Industrial methylated spirit	Hydroxyethyl cellulose
Carbomers	Sodium hydroxide
Diethylamine	Benzyl alcohol
Water	Isopropyl alcohol
	Water

## Data Availability

Not applicable.

## References

[1] Raney S.G., Luke M.C. (2020). A new paradigm for topical generic drug products: Impact on therapeutic access. J. Am. Acad. Dermatol..

[2] U.S. Department of Health & Human Services CFR-Code of Federal Regulations Title 21. https://www.accessdata.fda.gov/scripts/cdrh/cfdocs/cfcfr/CFRSearch.cfm?CFRPart=320&showFR=1.

[3] Grosser S., Park M., Raney S.G., Rantou E. (2015). Determining Equivalence for Generic Locally Acting Drug Products. Stat. Biopharm. Res..

[4] Narkar Y. (2010). Bioequivalence for Topical Products—An Update. Pharm. Res..

[5] Shah V.P., Flynn G.L., Yacobi A., Maibach H.I., Bon C., Fleischer N.M., Franz T.J., Kaplan S.A., Kawamoto J., Lesko L.J. (1998). Bioequivalence of Topical Dermatological Dosage Forms–Methods of Evaluation of Bioequivalence. Ski. Pharmacol. Physiol..

[6] Franz T.J., Lehman P.A., Raney S.G. (2009). Use of Excised Human Skin to Assess the Bioequivalence of Topical Products. Ski. Pharmacol. Physiol..

[7] Krishnaiah Y.S.R., Xu X., Rahman Z., Yang Y., Katragadda U., Lionberger R., Peters J.R., Uhl K., Khan M.A. (2014). Development of performance matrix for generic product equivalence of acyclovir topical creams. Int. J. Pharm..

[8] Lehman P.A., Raney S.G., Franz T.J. (2011). Percutaneous Absorption in Man: In vitro–in vivo Correlation. Ski. Pharmacol. Physiol..

[9] Skelly J.P., Shah V.P., Maibach H.I. (1987). FDA and AAPS report of the workshop on principles and practices of in vitro percutaneous penetration studies: Relevance to bioavailability and bioequivalence. Pharm. Res..

[10] Shin S.H., Rantou E., Raney S.G., Ghosh P., Hassan H., Stinchcomb A. (2020). Cutaneous Pharmacokinetics of Acyclovir Cream 5% Products: Evaluating Bioequivalence with an In Vitro Permeation Test and an Adaptation of Scaled Average Bioequivalence. Pharm. Res..

[11] US Food and Drug Administration Draft Guidance on Acyclovir. https://www.accessdata.fda.gov/drugsatfda_docs/psg/PSG_018604.pdf.

[12] Ghosh P., Raney S.G., Luke M.C. Evaluation of cutaneous pharmacokinetics: The past, the present and the future. Proceedings of the Visualizing and Quantifying Drug Distribution in Tissue V.

[13] Yacobi A., Shah V.P., Bashaw E.D., Benfeldt E., Davit B., Ganes D., Ghosh T., Kanfer I., Kasting G.B., Katz L. (2014). Current Challenges in Bioequivalence, Quality, and Novel Assessment Technologies for Topical Products. Pharm. Res..

[14] Caspers P.J., Lucassen G.W., Bruining H.A., Puppels G.J. (2000). Automated depth-scanning confocal Raman microspectrometer for rapid in vivo determination of water concentration profiles in human skin. J. Raman Spectrosc..

[15] Caspers P.J., Lucassen G.W., Wolthuis R., Bruining H.A., Puppels G.J. (1998). In vitro and in vivo Raman spectroscopy of human skin. Biospectroscopy.

[16] Mateus R., Abdalghafor H., Oliveira G., Hadgraft J., Lane M.E. (2013). A new paradigm in dermatopharmacokinetics–Confocal Raman Spectroscopy. Int. J. Pharm..

[17] Mohammed D., Matts P.J., Hadgraft J., Lane M.E. (2014). In Vitro–In Vivo Correlation in Skin Permeation. Pharm. Res..

[18] Caspers P.J., Nico C., Bakker Schut T.C., de Sterke J., Pudney P.D., Curto P.R., Illand A., Puppels G.J. (2019). Method to quantify the in vivo skin penetration of topically applied materials based on Confocal Raman Spectroscopy. Transl. Biophotonics.

[19] Iliopoulos F., Caspers P.J., Puppels G.J., Lane M.E. (2020). Franz Cell Diffusion Testing and Quantitative Confocal Raman Spectroscopy: In Vitro–In Vivo Correlation. Pharmaceutics.

[20] Patel A., Iliopoulos F., Caspers P.J., Puppels G.J., Lane M.E. (2021). In Vitro–In Vivo Correlation in Dermal Delivery: The Role of Excipients. Pharmaceutics.

[21] Patel A., Bell M., O’Connor C., Inchley A., Wibawa J., Lane M.E. (2013). Delivery of ibuprofen to the skin. Int. J. Pharm..

[22] The Medicines and Healthcare Products Regulatory Agency Public Assessment Report, UKPAR Ibuprofen 10% *w*/*w* Gel. https://mhraproducts4853.blob.core.windows.net/docs/170bdca5819db72058f65980fe718b14915b4693.

[23] European Medicines Agency List of Nationally Authorised Medicinal Products. https://www.ema.europa.eu/en/documents/psusa/ibuprofen-ibuprofen-lysine-not-indicated-ductus-arteriosus-ibuprofen/caffeine-list-nationally-authorised-medicinal-products-psusa/00010649/202002_en.pdf.

[24] Compendium E.M. Nurofen Maximum Strength 10% Gel. https://www.medicines.org.uk/emc/product/13002/smpc#gref.

[25] Compendium E.M. Ibuleve Maximum Strength Gel 50 g. https://www.medicines.org.uk/emc/product/8532/smpc#gref.

[26] ICH Harmonised Tripartite Validation of analytical procedures: Text and methodology Q2 (R1). Proceedings of the International Conference on Harmonization.

[27] Organization for Economic Cooperation Development (2004). Test No. 428: Skin Absorption: In Vitro Method.

[28] Organization for Economic Cooperation Development (2004). OECD Guidance Document for the Conduct of Skin Absorption Studies.

[29] Oliveira G., Hadgraft J., Lane M.E. (2012). The influence of volatile solvents on transport across model membranes and human skin. Int. J. Pharm..

[30] Andersen F.A. (1999). Final Report on the Safety Assessment of Oleth-2, -3, -4, -5, -6, -7, -8, -9, -10, -11, -12, -15, -16, -20, -23, -25, -30, -40, -44, and -501. Int. J. Toxicol..

[31] Caspers P.J., Bruining H.A., Puppels G.J., Lucassen G.W., Carter E.A. (2001). In Vivo Confocal Raman Microspectroscopy of the Skin: Noninvasive Determination of Molecular Concentration Profiles. J. Investig. Dermatol..

[32] Pradal J. (2020). Comparison of skin permeation and putative anti-inflammatory activity of commercially available topical products containing ibuprofen and diclofenac. J. Pain Res..

[33] UK Legislation The Methylated Spirits Regulations 1987. https://www.legislation.gov.uk/uksi/1987/2009/body/made.

[34] Watkinson R.M., Herkenne C., Guy R.H., Hadgraft J., Oliveira G., Lane M.E. (2009). Influence of Ethanol on the Solubility, Ionization and Permeation Characteristics of Ibuprofen in Silicone and Human Skin. Ski. Pharmacol. Physiol..

[35] Goh C.F., Boyd B.J., Craig D.Q.M., Lane M.E. (2020). Profiling of drug crystallization in the skin. Expert Opin. Drug Deliv..

[36] Goh C.F., Moffat J.G., Craig D.Q.M., Hadgraft J., Lane M.E. (2019). Monitoring Drug Crystallization in Percutaneous Penetration Using Localized Nanothermal Analysis and Photothermal Microspectroscopy. Mol. Pharm..

[37] Hadgraft J., Lane M.E. (2016). Drug crystallization–implications for topical and transdermal delivery. Expert Opin. Drug Deliv..

[38] Iliopoulos F., Goh C.F., Haque T., Rahma A., Lane M.E. (2022). Dermal Delivery of Diclofenac Sodium—In Vitro and In Vivo Studies. Pharmaceutics.

[39] Iliopoulos F., Caspers P.J., Puppels G.J., Lane M.E. Novel use of Confocal Raman Spectroscopy for in vivo quantification of skin permeants in real time: A non-invasive method for assessing bioequivalence. Proceedings of the Visualizing and Quantifying Drug Distribution in Tissue V.

[40] Mateus R., Moore D.J., Hadgraft J., Lane M.E. (2014). Percutaneous absorption of salicylic acid–in vitro and in vivo studies. Int. J. Pharm..

[41] Committee for Medicinal Products for Human Use, European Medicines Agency Guideline on the Investigation of Bioequivalence. https://www.ema.europa.eu/en/documents/scientific-guideline/guideline-investigation-bioequivalence-rev1_en.pdf.

[42] US Food and Drug Administration FDA Guidance for Industry Statistical Approaches to Establishing Bioequivalence. https://www.fda.gov/media/70958/download.

[43] US Food and Drug Administration Draft Guidance on Acyclovir. https://www.accessdata.fda.gov/drugsatfda_docs/psg/Acyclovir_topical%20cream_RLD%2021478_RV12-16.pdf.

[44] Yang Y., Ako-Adounvo A.-M., Wang J., Coelho S.G., Adah S.A., Matta M.K., Strauss D., Michele T.M., Wang J., Faustino P.J. (2022). In Vitro Testing of Sunscreens for Dermal Absorption: Method Comparison and Rank Order Correlation with In Vivo Absorption. AAPS PharmSciTech.

[45] Benfeldt E., Hansen S.H., Vølund A., Menné T., Shah V.P. (2007). Bioequivalence of Topical Formulations in Humans: Evaluation by Dermal Microdialysis Sampling and the Dermatopharmacokinetic Method. J. Investig. Dermatol..

[46] Bodenlenz M., Augustin T., Birngruber T., Tiffner K.I., Boulgaropoulos B., Schwingenschuh S., Raney S.G., Rantou E., Sinner F. (2020). Variability of Skin Pharmacokinetic Data: Insights from a Topical Bioequivalence Study Using Dermal Open Flow Microperfusion. Pharm. Res..

[47] van de Sandt J.J.M., van Burgsteden J.A., Cage S., Carmichael P.L., Dick I., Kenyon S., Korinth G., Larese F., Limasset J.C., Maas W.J.M. (2004). In vitro predictions of skin absorption of caffeine, testosterone, and benzoic acid: A multi-centre comparison study. Regul. Toxicol. Pharmacol..

